# Self-Oxygenation of Tissues Orchestrates Full-Thickness Vascularization of Living Implants

**DOI:** 10.1002/adfm.202100850

**Published:** 2021-07-06

**Authors:** Ali Farzin, Shabir Hassan, Liliana S. Moreira Teixeira, Melvin Gurian, João F. Crispim, Varun Manhas, Aurélie Carlier, Hojae Bae, Liesbet Geris, Iman Noshadi, Su Ryon Shin, Jeroen Leijten

**Affiliations:** Division of Engineering in Medicine Department of Medicine Brigham and Women’s Hospital Harvard Medical School Cambridge, MA 02139, USA; Department of Tissue Engineering and Applied Cell Sciences School of Advanced Technologies in Medicine Tehran University of Medical Sciences Tehran, Iran; Division of Engineering in Medicine Department of Medicine Brigham and Women’s Hospital Harvard Medical School Cambridge, MA 02139, USA; Department of Developmental BioEngineering Technical Medical Centre University of Twente Enschede, The Netherlands; Department of Developmental BioEngineering Technical Medical Centre University of Twente Enschede, The Netherlands; Department of Developmental BioEngineering Technical Medical CentreUniversity of Twente Enschede, The Netherlands; Biomechanics Research Unit GIGA In Silico Medicine University of Liège Chemin des Chevreuils 1, B52/3, Liège 4000, Belgium; Laboratory for Cell Biology-Inspired Tissue Engineering MERLN Institute University of Maastricht Maastricht, The Netherlands; KU Convergence Science and Technology Institute Department of Stem Cell and Regenerative Biotechnology Konkuk University Seoul 05029, Republic of Korea; Biomechanics Research Unit GIGA In Silico Medicine University of Liège Chemin des Chevreuils 1, B52/3, Liège 4000, Belgium; Department of Bioengineering University of California Riverside, CA 92521, USA; Division of Engineering in Medicine Department of Medicine Brigham and Women’s Hospital Harvard Medical School Cambridge, MA 02139, USA; Division of Engineering in Medicine Department of Medicine Brigham and Women’s Hospital Harvard Medical School Cambridge, MA 02139, USA; Department of Developmental BioEngineering Technical Medical Centre University of Twente Enschede, The Netherlands

**Keywords:** angiogenesis, calcium peroxide, cellular metabolism, hydrophobic micromaterials, implant survival, oxygen generation

## Abstract

Bioengineering of tissues and organs has the potential to generate functional replacement organs. However, achieving the full-thickness vascularization that is required for long-term survival of living implants has remained a grand challenge, especially for clinically sized implants. During the pre-vascular phase, implanted engineered tissues are forced to metabolically rely on the diffusion of nutrients from adjacent host-tissue, which for larger living implants results in anoxia, cell death, and ultimately implant failure. Here it is reported that this challenge can be addressed by engineering self-oxygenating tissues, which is achieved via the incorporation of hydrophobic oxygen-generating micromaterials into engineered tissues. Self-oxygenation of tissues transforms anoxic stresses into hypoxic stimulation in a homogenous and tissue size-independent manner. The in situ elevation of oxygen tension enables the sustained production of high quantities of angiogenic factors by implanted cells, which are offered a metabolically protected pro-angiogenic microenvironment. Numerical simulations predict that self-oxygenation of living tissues will effectively orchestrate rapid full-thickness vascularization of implanted tissues, which is empirically confirmed via in vivo experimentation. Self-oxygenation of tissues thus represents a novel, effective, and widely applicable strategy to enable the vascularization living implants, which is expected to advance organ transplantation and regenerative medicine applications.

## 1. Introduction

The bioengineering of tissues and organs has the potential to deliver functional replacement organs.^[1,2]^ However, the creation of viable clinically sized implants has remained a grand challenge. Current tissue engineering strategies, although suitable for the development of small living tissues that remain viable upon implantation, are incompatible with maintaining the viability of larger implants. Specifically, while small implants can rely on host-to-implant diffusion of oxygen and nutrients, large implants inevitably suffer from nutrient diffusion limitations that induce anoxia-induced cell death and sub-sequent implant failure.^[3,4^]

Various strategies have been explored to accelerate the implant’s access to the host’s oxygen, metabolites, and nutrients. These strategies commonly rely on endowing implants with angiogenic growth factors, (pro)angiogenic cells, and/or bioprinting of vascular structures to establish functional anastomosis.^[5–7]^ Although successful in small implants, these angiogenic and vasculogenic strategies are ineffective for larger implants due to the required fundamental biological processes that impose absolute limits on the rate in which functional blood vessels can be established within implants, which amongst other includes the migration speed and proliferation rate of endothelial cells. Moreover, incorporation of angiogenic cells or growth factors will further increase the burden of an already metabolically deprived tissue by having to support additional cells as well as the angiogenic process.^[8,9]^ Furthermore, before achieving functional anastomosis, implanted engineered tissues will be rapidly depleted of oxygen resulting in hypoxic (<5% dissolved oxygen) followed by anoxic (<0.5% dissolved oxygen) microenvironments.^[10,11]^ While acute hypoxia is conducive for regenerative responses such as angiogenesis, anoxia inhibits these regenerative processes and causes cell death.^[12]^ A living implant’s ability to vascularize and survive is therefore inversely correlated with the duration and intensity of its metabolic deprivation, which currently is dictated predominantly by the implant’s size.^[13]^ Consequently, the historically explored metabolism-dependent pro-angiogenic and vasculogenic strategies have not been able to offer an effective solution to orchestrate full-thickness vascularization of clinically sized living implants.^[14]^

We hypothesized that the incorporation of a controlled oxygen release system into a living implant would facilitate its vascularization by transforming the cytotoxic anoxia into pro-angiogenic hypoxia. Advantageously, the proposed metabolic support is only transiently required as it is designed to bridge the implant’s prevascular period. To achieve this feat, a system for the controlled and prolonged release of oxygen is needed. Several oxygen releasing and generating strategies have already been developed, for example, hemoglobin and myoglobin sub-stitutes, polymer-based oxygen carriers, perfluorocarbons, and solid peroxides.^[15–19]^ These compounds and materials have the ability to bind, dissolve, or generate physiologically relevant quantities of oxygen, but typically release their payloads within minutes to hours. In contrast, alleviating the oxygen deprivation during the prevascular phase of large implants demands release periods of multiple days to weeks.^[20,21]^ Interestingly, week-long oxygen generation could potentially be achieved by encapsulating a solid peroxide such as calcium peroxide (CaO_2_; CPO) in hydrophobic bulk material.^[22]^ Specifically, a material’s hydrophobic nature could be used to limit the exposure of encapsulated solid peroxides to water molecules, which effectively grants control over the hydrolysis rate of solid peroxides and hence oxygen release. Unfortunately, encapsulating cells in hydrophobic materials associates with poor outcomes in terms of cell survival and tissue formation. In short, the development of biocompatible long-term oxygen generating biomaterial capable of sustaining the survival and function of clinically sized tissues has remained a challenge.

Here, we report the development of novel micromaterials that function as hydrophobic oxygen-generators (HOGs), which can be readily integrated within engineered tissues. HOGs allowed for safe and long-term oxygen release that granted control over the in situ oxygen tension by alleviating anoxic stress throughout the entire volume of the engineered tissues. Most notably, the incorporation of HOGs orchestrated the rapid vascularization of implanted living tissues. Specifically, metabolic support enabled the survival of encapsulated stem cells, which facilitated the production of high quantities of vascular endothelial growth factor (VEGF). Uniquely, we here report that mildly increasing the oxygen tension in implanted tissues drives full-thickness vascularization of living implants. HOGs therefore represent a promising solution to maintain the survival of clinically sized engineered tissues and facilitate their functional integration within the receiving host.

## 2. Results

### 2.1. Fabrication and Characterization of HOGs

The stoichiometry of CPO’s oxygen generating ability is described by the following chemical equations:

(1) $${\text{CaO}}_{2}+2{\text{H}}_{2}\text{O}\to \text{Ca}{\left(\text{OH}\right)}_{2}+{\text{H}}_{2}{\text{O}}_{2}$$

(2) $$2{\text{H}}_{2}{\text{O}}_{2}\to {\text{O}}_{2}+2{\text{H}}_{2}\text{O}$$

**Figure 1.**
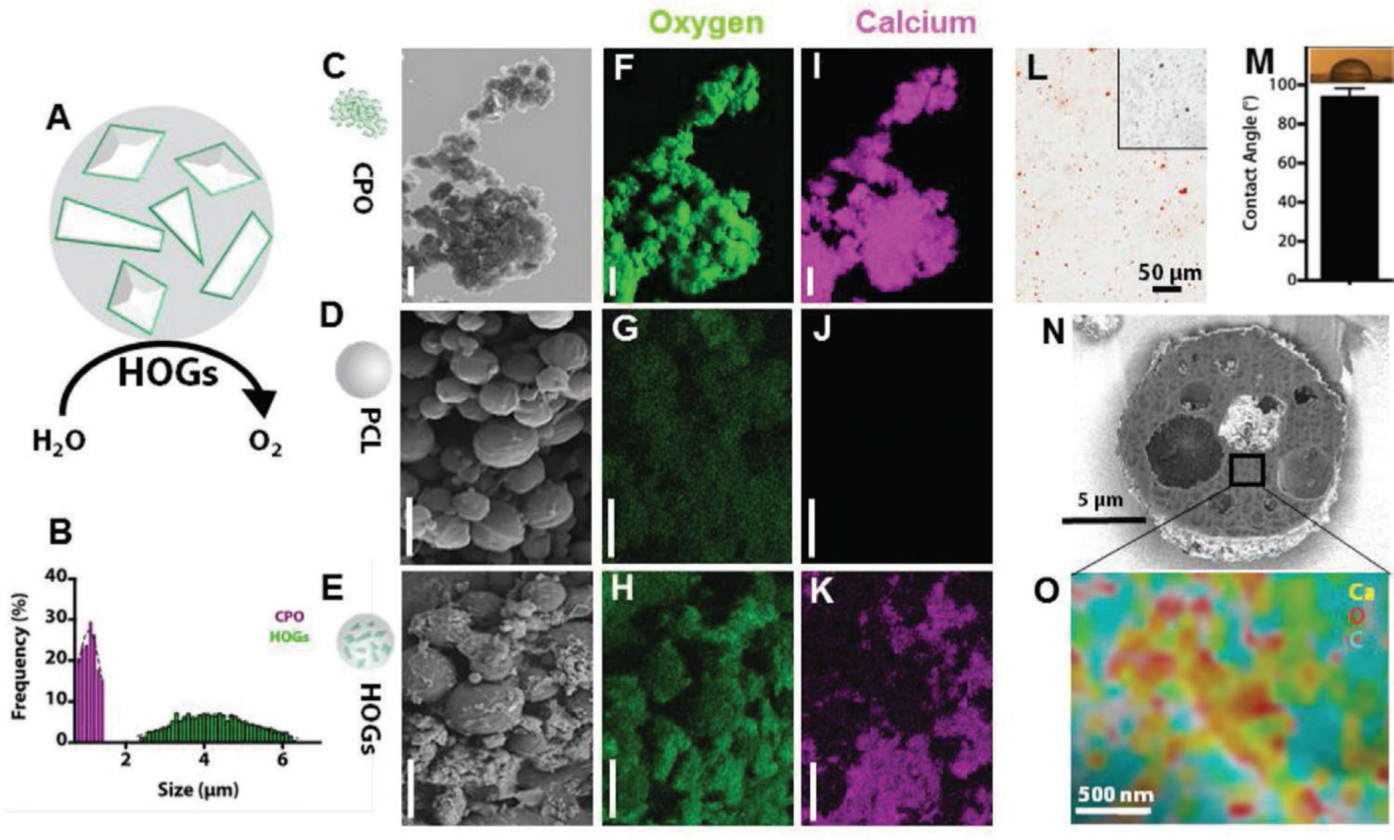
Production of HOGs via incorporation of oxygen generating compounds in hydrophobic microparticles. A) Schematic illustration of HOGs in which CPO’s exposure to water penetration is limited based on the PCL microparticle’s hydrophobic nature. B) DLS particle size distribution of CPO and HOGs (*n* = 3). C–K) SEM microphotographs and elemental mapping of oxygen and calcium by EDS of the coincident surface of CPO, PCL, and HOGs. Scale bars equal 4 μm. L) Microphotograph of Alizarin Red S stained HOGs and pristine PCL microparticles (inset). M) WCA measurement of HOGs coated surface. (*n* = 3). N) SEM microphotograph and O) EDS image of HOGs that were cut in the midsagittal plane using a FIB.

From these equations, it is evident that oxygen release from CPO can thus be rate limited by controlling the exposure of CPO to water molecules. We, therefore, theorized that the encapsulation of CPO in hydrophobic microparticles—named HOGs—would result in a controlled and long-term oxygen release system (Figure 1A). HOG microparticles were produced using a controllable and scalable water-in-oil-in-water double emulsion synthesis method. Pristine polycaprolactone (PCL) micromaterials were synthesized as a non-oxygen generating control. Size analysis revealed that PCL MPs, pristine CPO, and HOGs associated with a diameter of 2.3 ± 0.9, 1.2 ± 0.2, and 4.6 ± 1.2 μm, respectively (Figure 1B).

Varying the final CPO concentration within HOGs did not measurably affect the size of the synthesized microparticles. With an encapsulation efficiency of >90%, CPO in concentrations up to 20% (wt./wt.) did not significantly affect the encapsulation efficiency and CPO loading capacity in HOGs (Figure S2, Supporting Information). Micromorphological analysis of CPO, PCL, and HOGs by scanning electron microscopy (SEM) confirmed the spherical and intact nature of PCL and HOG micromaterials (Figure 1C–E). Energy dispersive X-ray spectrometry (EDS) based elemental mapping suggested relatively higher oxygen content in HOGs as compared to pristine PCL micromaterials (Figure 1F–H). Incubating HOGs under standard culture conditions for twelve days resulted in a notable decline in relative oxygen content in HOGs, which suggested the release of oxygen from the HOGs into the culture medium (Figure S3, Supporting Information). Moreover, elemental mapping confirmed the presence of calcium in HOGs, which corroborated the presence of CPO in HOGs as pristine PCL micromaterials did not contain any detectable levels of calcium (Figure 1I-K). Indeed, Alizarin Red S stained HOGs and not pristine PCL microparticles (Figure 1L). The hydrophobic nature of PCL was confirmed via water contact angle (WCA) measurements, which was diminished in a dose-dependent manner following CPO incorporation (Figure 1M and Figure S4, Supporting Information). The pristine PCL polymer is hydrophobic in nature. PCL sheets exhibited WCA of 94 ± 4° and water drops could remain on the surface of PCL for a long time (Figure S4, Supporting Information EDX). The incorporation of CPO in PCL polymer showed a significant decrease in WCA pointing toward reduced hydrophobicity and thus increased hydrophilicity. As the hydrophobicity of PCL was inversely correlated with the concentration of CPO particles, the presence of CPO on the surface of HOGs was assumed. To confirm that CPO was efficiently encapsulated within the PCL microparticles, HOGs were cut open using a focused ion beam (FIB) and imaged using SEM, which revealed homogeneously distributed darker regions within the HOGs that matched CPO’s particle size (Figure 1N). High-resolution elemental mapping of FIB-SEM cut HOGs confirmed that these regions were CPO nanoparticles based on the spatial distribution patterns of calcium, oxygen, and carbon (Figure 1N). Indeed, the vast majority of CPO was detected within the bulk of the HOGs.

### 2.2. Engineering Self-Oxygenating Hydrogels using HOGs

**Figure 2.**
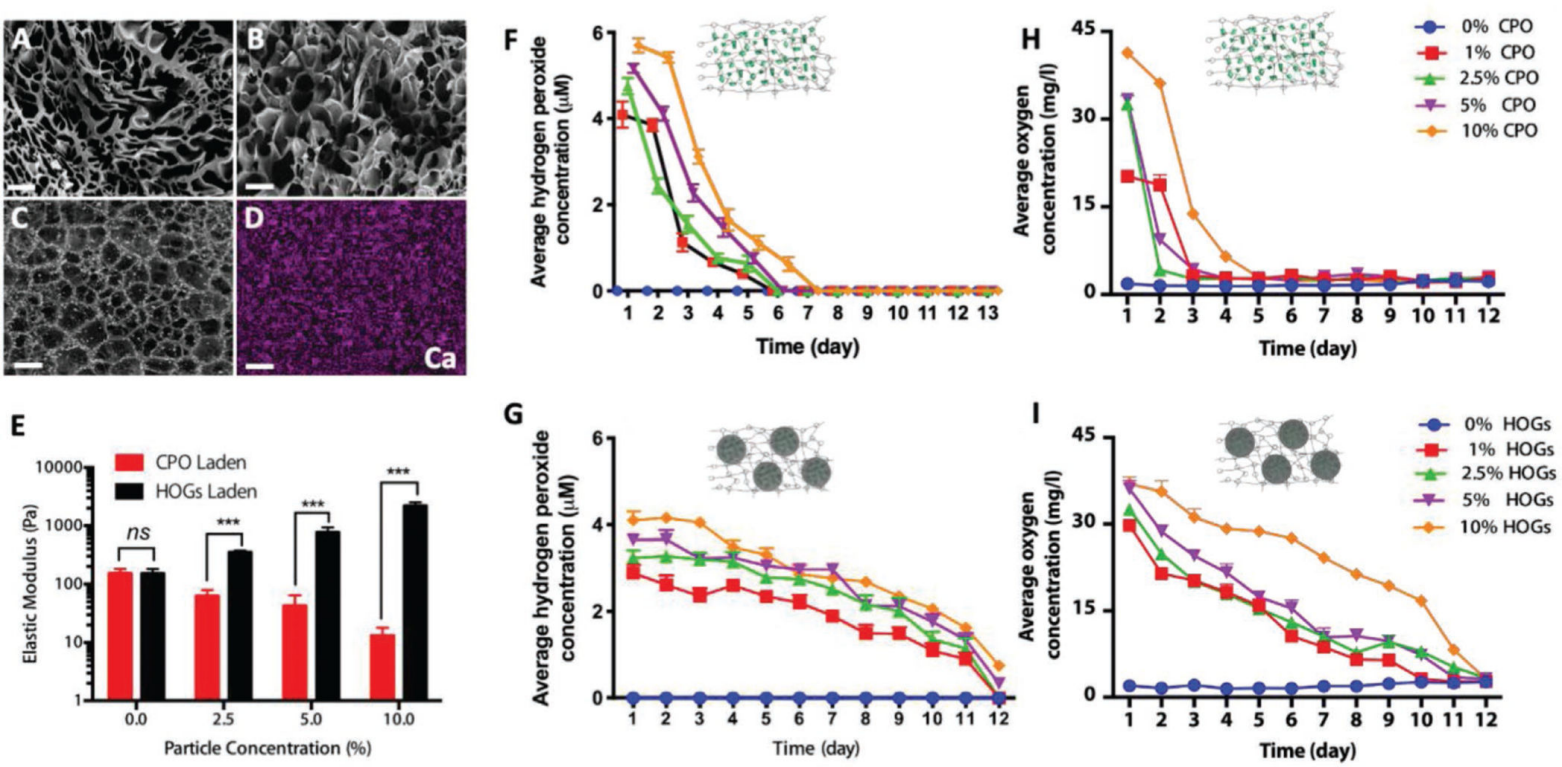
Encapsulation of HOGs in photocrosslinked biomaterials allows for long-term release of oxygen in 3D engineered constructs. SEM microphotographs of freeze-dried A) GelMA, B) GelMA containing 20% (wt./vol.) of CPO, and C) GelMA containing 20% wt./vol.) of HOGs. D) Elemental mapping of calcium by EDS of a GelMA hydrogel containing 20% (wt./vol.) of HOGs. E) Elastic modulus of GelMA hydrogels containing various concentrations of CPO and HOGs. F,G) Hydrogen peroxide and H,I) oxygen release kinetics of self-oxygenating hydrogels containing different concentrations of F,H) CPO and G,I) HOGs under severe hypoxic condition. Significant differences are shown as *p < 0.05, **p < 0.01, and ***p < 0.001. Scale bars equal 100 μm. *n* = 3 for all experiments.

HOGs were used to create a novel class of self-oxygenating hydrogels. Specifically, HOGs containing various CPO concentrations (e.g., 0%, 1%, 2.5%, 5%, and 10% (wt./vol.)) were mixed in gelatin methacryloyl (GelMA) polymer solutions resulting in self-oxygenating hydrogel, which were photocrosslinked in molds to form 4 mm wide and 1 mm thick cylindrical multiphase polymeric microcomposites. As GelMA is composed of gelatin, an amphiphilic polymer that has shown excellent properties as a stabilizer for drug delivery and tissue engineering applications it was expected to allow for homogeneous dispersion of HOGs with GelMA hydrogels.^[23]^ Realizing a homogenous distribution of HOGs in the hydrophilic bulk material is essential to achieve consistent and equally distributed oxygenation of engineered materials. Homogeneous distribution CPO or HOGs particles was demonstrated in freeze-dried GelMA hydrogel constructs using SEM analysis (Figure 2A–C), which was corroborated using EDS based spatial mapping of calcium (Figure 2D). Incorporation of pristine CPO dose-dependently increased the swelling of the hydrogel and decreased its overall elastic modulus (Figure 2E and Figure S5, Supporting Information). The higher swelling ratio of GelMA hydrogel containing CPO, as compared to those loaded by same amount of HOGs (Figure S5, Supporting Information), suggests that the hydrophilic nature of CPO and the porosities of hydrogel structure had an effect on the absorption and retention of aqueous media, respectively. The porosities of GelMA with 20% CPO (wt./vol.) and GelMA with 20% HOGs (wt./vol.) were analyzed for Figure 2B,C by ImageJ software and calculated to be 83 ± 2.6% and 74 ± 0.8%, respectively. This could potentially be explained by the intense burst release of O_2_ producing micro-bubbles that cause a more porous structure in case of CPO containing hydrogel as compared to a hydrogel containing HOGs. This might also partially explain the difference in the mechanical properties between these hydrogels (Figure 2E). This was most likely mediated via the quenching of the photoexcited photoinitiator via the fast-paced generation of oxygen of non-encapsulated CPO.^[24]^ Indeed, incorporation of HOGs minimized the swelling compared to the GelMA hydrogel with CPO only, albeit, without decreasing the hydrogel’s elastic modulus. In fact, the elastic modulus increased in a dose-dependent manner becoming over an order of magnitude higher for 10% HOG-GelMA as compared to pristine GelMA. Previous reports of multiphase polymeric microcomposites in which polymeric micromaterials where incorporated in hydrogel systems have made similar observations.^[25,26]^ Moreover, this avoidance of decrease in elastic modulus suggested that HOGs indeed mitigated the initial bulk release of oxygen by reducing the rate of the hydrolytic conversion of CPO into calcium hydroxide and hydrogen peroxide. To validate this, hydrogen peroxide release kinetics of CPO-GelMA and HOG-GelMA were determined for up to twelve days. These release kinetics are in line with CPO being exposed directly to water and are in agreement with previous literature.^[17]^ In contrast, HOG-GelMA hydrogels released notably lower levels of hydrogen peroxide at initial stages, which slowly and stably declined over time (Figure 2G). HOGs thus offer cytoprotection from the oxidative stresses by partially shielding cells from hydrogen peroxide-induced toxicity. As the hydrogen peroxide is subsequently converted to oxygen and water, the oxygen concentration in CPO-GelMA and HOG-GelMA hydrogels was determined in a time-resolved manner inside a continually purged modular hypoxia incubator. While CPO-GelMA hydrogels associated with a short-lived oxygen generation, HOG-GelMA hydrogels were characterized by a sustained release of oxygen for nearly two weeks (Figure 2H,I). Although the CPO or HOG concentration did not have a major effect on the release duration of hydrogen peroxide or oxygen, it positively correlated with the released quantities. Consequently, varying the CPO concentration allowed for tuning of the oxygen concentration without affecting its release duration. Overcoming diffusion limits for survival of any cellularized implanted tissue,^[27]^ the observed oxygen release kinetics of HOGs-GelMA were seen to be in line with those required to bridge the prevascular phase survival of living implants.^[28–30]^

### 2.3. Self-Oxygenating Tissues Survive and Express High Levels of VEGF under Anoxia

**Figure 3.**
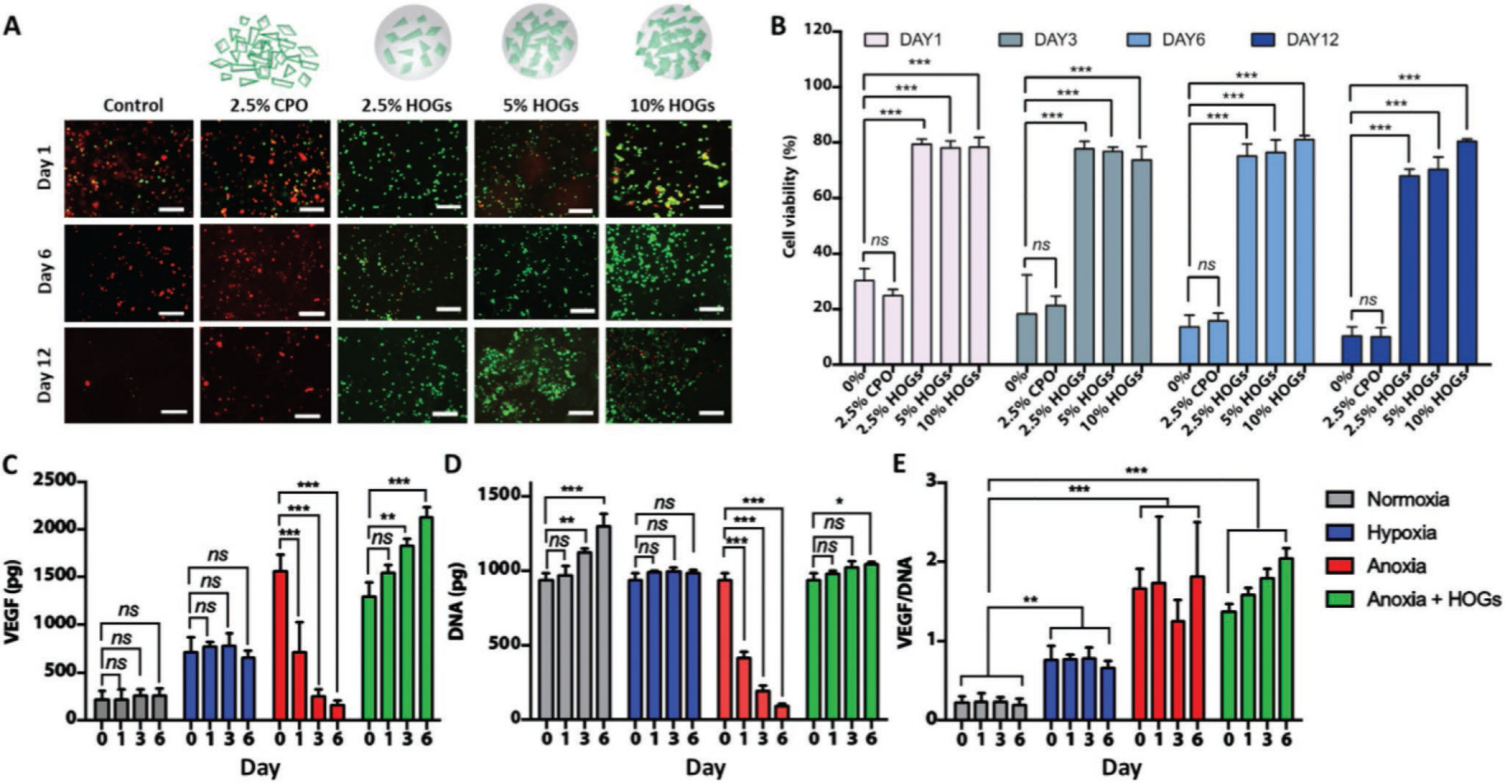
Self-oxygenation of tissues enables continued cell survival and VEGF expression under anoxic conditions. A) Confocal fluorescent microscopic images of self-oxygenating tissues composed of MSCs in GelMA hydrogel and CPO or HOGs that were cultured under anoxic conditions and stained with calcein-AM (live:green)/ethidium homodimer (dead:red). B) Semi-quantification of MSCs viability of self-oxygenating tissues cultured under anoxic culture conditions (*n* = 7). C) Total amount of secreted VEGF (*n* = 3) and D) DNA were quantified and used to determine (*n* = 3) E) VEGF/DNA ratio of non-oxygen generating tissues composed of hMSCs encapsulated GelMA hydrogels that were cultured under normoxic, hypoxic, and anoxic conditions and oxygen generating tissues composed of 2.5% HOG-GelMA that were cultured under anoxic conditions (*n* = 3). Scale bars equal 100 μm. Significant differences are shown as *p < 0.05, **p < 0.01, and ***p < 0.001.

The ability of hydrophobic micromaterials to protect cells from the cytotoxic effects of the generated hydrogen peroxide was investigated. Self-oxygenating tissues were produced by encapsulating human mesenchymal stem cells (hMSCs) in GelMA hydrogels containing up to 10% of CPO or HOGs. It was observed that under normoxic conditions the addition of CPO to GelMA resulted in massive cell death, even at concentrations as low as 2.5% CPO (Figure S7A,B, Supporting Information). In contrast, self-oxygenating tissues based on HOG incorporation demonstrated only a minor and transient decrease in cell survival, even at concentrations of HOGs as high as 10% (Figure S7C,D). This vast improvement in cell survival confirmed HOGs’ cytoprotective capacity, which was most likely achieved by rate-limiting the buildup of hydrogen peroxide. We next determined whether HOGs also offered cytoprotection in terms of cell survival under anoxic culture conditions by metabolically supporting the encapsulated cells. Control tissues cultured under anoxic culture conditions rapidly and progressively reduced the percent of viable cells: after a single day only <30% of cells survived, which further declined to <10% after 12 days of culture (Figure 3). CPO-based self-oxygenating tissues associated with further decreased cell survival rates, which was most likely caused by the rapid generation of hydrogen peroxide. In fact, virtually none of the encapsulated hMSCs remained viable after six days of anoxic culture (Figure S8, Supporting Information). Importantly, self-oxygenating tissues based on HOG-GelMA formulations enabled ≈80% cells to remain viable over a period of 12 days under the hostile anoxic culture conditions, even at concentrations as low as 2.5% HOGs (Figure 3A,B). This survival rate of all engineered tissues was near-identical regardless of anoxic or normoxic culture condition, which indicated that HOGs could effectively avoid anoxia-induced cell death throughout the entire volume of self-oxygenating tissues. We then reasoned that the ability of HOGs to engineer consistently hypoxic microenvironments could be leveraged to stimulate encapsulated cells to express physiologically relevant levels of angiogenic factors such as VEGF. To test this hypothesis, self-oxygenating tissues composed of hMSCs encapsulated in 2.5% HOG-GelMA cultured under anoxic conditions were compared to HOG-free engineered tissues cultured under various oxygen tensions (e.g., normoxic, hypoxic, and anoxic). As expected, the culture’s oxygen concentration was inversely correlated with the total amount of secreted VEGF (Figure 3C). However, the total amount of VEGF secreted by control (e.g., non-oxygenating) tissues cultured under anoxic conditions progressively dwindled over time to levels even below those produced by control tissues cultured under normoxic conditions. In contrast, self-oxygenating tissues consistently produced high levels of VEGF over a period of six days. This remarkable difference between self-oxygenating tissues and control tissues could be explained by a progressive cell loss in control tissues caused by the anoxic culture conditions (Figure 3D). Indeed, the amount of VEGF secreted per cell had remained consistently high in control tissues despite the cell loss (Figure 3E), which was in line with previous reported literature.^[31,32]^ However, the angiogenic behavior of the implant is not determined by the per cell production of angiogenic factors, but rather via the formation of chemotactic gradients based on the total amount of angiogenic factors. Consequently, we hypothesized that implanted self-oxygenating tissues would demonstrate improved angiogenic behavior as compared to their conventional counterparts.

### 2.4. HOG Mediated Self-Oxygenation of Engineered Tissues Orchestrates Full-Thickness Vascularization In Vivo

**Figure 4.**
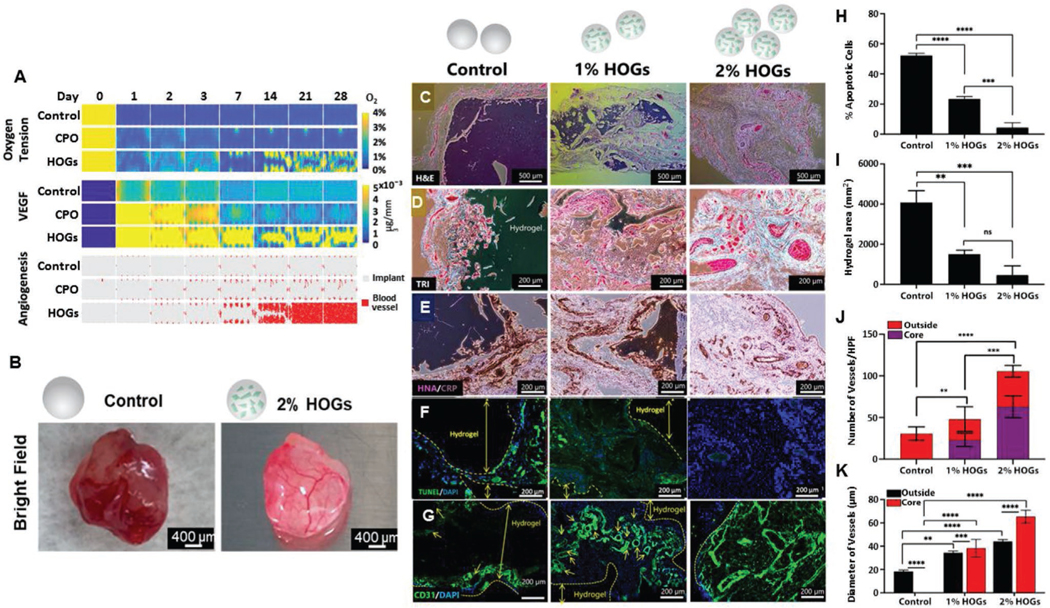
Self-oxygenation of tissues drives full-thickness vascularization. A) The simulated numerical model of oxygen tension, VEGF release, and angiogenesis in non-oxygen generating tissues and oxygen generating tissues containing either 2% (wt./vol.) of CPO or 2% (wt./vol.) of HOGs following virtual implantation. GelMA hydrogels containing 2*10^6^ cells mL^−1^ of hMSCs and 0%, 1%, or 2% of HOGs were subcutaneously implanted in rats for seven days. B) Bright-field images of explants were taken seven days post-implantation. Microphotographs of 5 micrometer thin midsaggital section of the explants that were stained with C) H&E, D) TRI, E) HNA/CRP, F) TUNEL/DAPI, and G) CD31/DAPI. Semi-quantitative image analysis of core and outside of stained tissue sections was performed to evaluate the correlation between the HOG concentration and H) % of apoptotic cells, I) degradation rate of implanted hydrogels, J) number of formed vessels, and K) diameter of formed vessels. Significant differences are shown as **p < 0.05, ***p < 0.001, and ****p < 0.0001.

To predict the in vivo behavior of self-oxygenating tissues, numerical simulations were performed to model their behavior following their implantation. Specifically, a previously developed set of partial differential equations of the taxis-diffusion-reaction type were used to describe various key cellular processes including oxygen tension fluctuations, angiogenic factor production, angiogenesis, cell proliferation, and cell death.^[33]^ This model has previously been used to corroborate that oxygen tension was a key factor in terms of implant failure in clinically sized tissues.^[34]^ Here, we have adapted the numerical model by incorporating the approximated oxygen generation kinetics of 1%, 2%, and 4% of CPO or HOGs wt./v. into the simulated tissue implant (Figure S9, Supporting Information). In the control tissues, the simulated oxygen tension rapidly dropped to anoxic levels, which predicted poor VEGF production and angiogenesis (Figure 4A). Although CPO incorporation elevated the oxygen tension substantially during the first 24 h following implantation, the simulation predicted a neglectable improvement in implant vascularization due to oxygen depletion after three days of implantation. In contrast, simulated self-oxygenating tissue implants effectively prevented the formation of anoxic regions, which maintained cell survival and resulted in potent VEGF production. This associated with improved and accelerated vascularization that enabled full-thickness vascularization of the simulated self-oxygenating tissues. In addition, increasing the HOG concentration to 4% was predicted to have little additional effects while reducing the HOG concentration to 1% would mitigate the beneficial effect (Figure S10A,B, Supporting Information). To empirically validate these simulations driven predictions, engineered tissues composed 50 mm^3^ GelMA hydrogels containing 2×10^6^ cells mL^−1^ hMSCs and 0%, 1%, or 2% HOGs were subcutaneously implanted in rats for seven days. The macroscopic analysis revealed a striking difference in gross appearance between the explanted tissues. Where control tissues were characterized by a dark color that was reminiscent of necrotic tissue, self-oxygenating tissues possessed a healthy-looking light-pink appearance with complex patterns that suggested vascular structures (Figure 4B). Whole explant live/dead analysis confirmed that while control tissues were mostly void of living cells, self-oxygenating tissue were still populated by numerous living cells and appeared to indeed possess intricate networks of vessels. To study host–graft interaction, midsagittal sections of the explants were analyzed using histology and immunohistochemistry. Hematoxylin and eosin (H&E) (Figure 4C) and Masson’s trichrome (TRI) (Figure 4D) staining confirmed that the core of control tissues was void of cells. Moreover, control tissues demonstrated low levels of biomaterial degradation, absence of vessels, and contained cellular infiltration in their peripheral regions, which were covered in a fibrous capsule. In marked contrast, self-oxygenating tissues contained a high number of cells, lacked a fibrous capsule, and possessed an abundance of vessel-like structures throughout the entire implant. We postulated that these distinct differences between conventional and self-oxygenating tissues could be explained by the retention or loss of hMSCs and their associated bioactivity, immunoregulatory, and tissue remodeling properties. Indeed, Human nuclear antigen (HNA) staining confirmed that the hMSCs of self-oxygenating tissue remained present in the implant and were predominantly located around the vessels (Figure 4E). In addition, while control tissues displayed an intense C-reactive protein (CRP) staining at their peripheral regions, self-oxygenating tissues presented notably milder level. This reduction in local inflammation could potentially have contributed to the improved survival and retention of the implanted hMSCs. Tunel staining confirmed that locally inflamed regions indeed associated with increased levels of apoptosis, which was present at a notably lower level in self-oxygenating tissues containing 2% of HOGs (Figure 4F). Self-oxygenation of tissues also appeared to contribute to the integration with host tissue as the observed vessels were HNA-and CD31+, which suggested ingrowth of the host’s vascular system (Figure 4G). While control tissues exclusively presented CD31+ vessels in their peripheral regions, self-oxygenating tissues contained CD31+ vessels throughout their volume. Semi-quantification of stained tissue sections corroborated the inverse correlation between the HOG concentration and the hydrogel degradation as well as the number of apoptotic cells (Figure 4H,I). In line with these findings, the HOG concentration positively correlated with an increase in the number of CD31+ vessels, especially those located at the core of the implant (Figure 4J). HOGs also contributed to blood vessel maturation within the living implant in terms of vessel diameter (Figure 4K). HOGs thus enabled full-thickness implant vascularization, which was most likely mediated via the VEGF secreted by metabolically supported hMSCs.

## 3. Discussion

We here report on HOG micromaterial mediated self-oxygenation of engineered tissues, which enabled the survival and full-thickness vascularization of implanted tissues. Specifically, HOGs alleviated anoxic stress throughout the bulk of engineered tissues over a prolonged period of time, which allowed the tissue implant to survive independently of the oxygen that diffused from the host organism.

Encapsulating hydrophilic CPO in hydrophobic PCL endowed the resulting HOGs with an amphiphilic nature, albeit, with dominant hydrophobic characteristics owing to their PCL bulk. This composite based micromaterial design extended the release of oxygen-derived from CPO hydrolysis by several folds as compared to pristine CPO. While the hydrophobic nature of PCL slows down hydrolysis, PCL’s relatively high oxygen permeability coefficient allows for the near-instant release of generated oxygen to surrounding cells and tissues.^[35]^ HOGs generated sufficient amounts of oxygen in the implanted tissues to transform their own milieu into a hypoxic microenvironment, which enabled control over the living implant’s fate. HOG hydrophobicity can thus be tuned to control CPO hydrolysis and hence determine the rate and duration of oxygen release to cater to the physiologic needs of the tissue of choice. Matching oxygen generation with oxygen consumption is essential to orchestrate implant vascularization as both anoxic and normoxic microenvironments are detrimental to implant vascularization due to massive cell death and low-level production of angiogenic growth factors, respectively. In contrast, we aimed for the engineering of hypoxic microenvironments that allowed for continued cell survival and production of high levels of angiogenic factors, which was achieved using HOGs and associates with rapid full-thickness implant vascularization.

Self-oxygenation of engineered tissues via CPO is achieved by generating oxygen via the intermediate generation of hydrogen peroxide, which can induce cytotoxicity. Fortunately, PCL has been shown to shield cells from H_2_O_2_ insults up to 150 μm, which represents concentrations many times higher than the levels observed in our study. CPO is known to adversely impact cell survival when present at a concentration above 3% wt./vol.^[36]^ This is most likely caused by the resulting change in pH, an increase in calcium ions, or an increase in free radicals. We observed this phenomenon with CPO, but not with HOGs, which remained cytocompatible at substantially higher concentrations.

A unique feature of this work is the demonstration that self-oxygenation of tissues orchestrates full-thickness vascularization following implantation via in situ control over implant metabolism. This metabolism supporting strategy distinguishes this work from previous research, which has focused on strategies that are associated with an increase in metabolic burden (e.g., incorporation of angiogenic cells, vasculogenic cells, or growth factors).^[37]^ Although metabolically burdening approaches can be effective in small implants, they are ineffective for larger implants. Specifically, implanted clinically sized solid tissues will inherently develop large anoxic and metabolically deprived regions, which not only induce the death of implanted cells, but also thwart the host’s angiogenesis processes. We here introduce the concept of homogenous self-oxygenation of living implants to offer both short-term (e.g., cell survival) and long-term (full-thickness implant vascularization) protection by metabolically supporting implanted living tissues using HOGs to engineer stably hypoxic microevironments that produce high levels of angiogenic factors such as VEGF. Although the vascularization observed in this study was primarily achieved via angiogenesis, HOGs’ ability to sustain cell survival while driving the production of high levels of VEGF also has the potential to improve implant vascularization for tissue engineering strategies that aim to utilize vasculogenesis. Elevating the local oxygen tension of an implant to hypoxic levels for a prolonged period of time via self-oxygenation thus allows for the successful bridging of an implant’s prevascular period, which has remained a key hurdle in translating engineered tissue into a clinical reality. In particular, self-oxygenation driven vascularization of tissues is particularly suited for applications that require the implantation of a living implant of voluminous sizes with intense and/or intense vascularization of the implant. Such applications include, but are not limited to, engineered tissues to address critically sized bone defects, volumetric muscle loss, kidney failure, chronic liver disorders, and various cardiac pathologies.

While a working understanding of how metabolic programming can steer cell fate has been gained in recent years, tools to accurately control the metabolism (e.g., by controlling the oxygen tension for prolonged period of time) in vivo have remained scarce.^[38,39]^ HOGs, therefore, represent a novel enabling tool to steer the behavior of implanted living tissues. Moreover, this work underlines the relevance of including metabolic parameters into the design space of engineered tissues, which has remained an underexplored domain. Indeed, in situ control over the oxygen tension can guide the formation and maturation of engineered tissues via metabolic programming of cell fate.^[40,41]^ It is of note that the current study is limited to the use of PCL, however, it is anticipated that alternative hydrophobic materials will also offer extended oxygen generation profiles owing to their ability to limit calcium peroxide hydrolysis mediated homolytic cleavage.

The current study has explored the use of HOGs to create self-oxygenating tissues to orchestrate angiogenesis and enable full-thickness vascularization of living implants. However, HOGs could offer potential solutions for numerous additional challenges. Besides supporting the survival of implanted cells, HOGs can also steer the function and fate of implanted cells and host cells that are in close proximity to the implant via metabolic programming by controlling the local oxygen tension. Moreover, HOGs ability to control the oxygen tension in vivo could also be explored to treat pathologies that are adversely affected by prolonged hypoxia such as diabetic ulcers, tumors, and asphyxial cardiac arrest.

## 4. Conclusion

In this study, we demonstrated that oxygen generating micromaterials can be leveraged to improve the vascularization of living implants. Specifically, microencapsulation of oxygen releasing material such as solid peroxides allows for prolonged oxygen release duration while simultaneously minimizing the cytotoxicity of oxygen generation process. We demonstrated that pristine CPO in cellularized hydrogels is toxic due to the nature of CPO hydrolysis that associates with an intense rapid burst release of hydrogen peroxide and oxygen, which lasts only for a few days. In contrast, microencapsulation of solid peroxides within a hydrophobic material lowered the hydrogen peroxide concentration and extended the duration of oxygen generation. HOGs were demonstrated to allow for self-oxygenation of tissues to alleviate the detrimental anoxic stresses within the cellular constructs by creating a pro-angiogenic hypoxic microenvironment, which associated with production of high levels of VEGF over a prolonged period of time owing to continued cellular survival. In vivo experimentation demonstrated that self-oxygenation of engineered living tissues potently orchestrated rapid full-thickness vascularization and offered control over the in vivo oxygen tension for prolonged periods of time, which thus offered a method to metabolically program the fate and behavior of both implant and host. HOGs therefore represent a promising stepping-stone toward the development of clinically sized tissues for regenerative applications.

## 5. Experimental Section

### Preparation of CPO-Laden PCL Microparticles:

A double emulsion synthesis method of water-in-oil-in-water (w/o/w) was used to prepare CPO-laden PCL microparticles. Briefly, 3 mL of 10% (wt./vol.) PCL (Mw = 80 000, Sigma Aldrich) solution was prepared in dichloromethane (DCM, CH_2_Cl_2_, purity ≥99.8%, Sigma Aldrich). To obtain 0%, 5%, 10%, 15%, 20%, and 30% (wt./wt.) CPO in PCL microparticles, the predetermined concentration of CPO (CaO_2_, Sigma Aldrich) in 1 mL of ethanol (CH_3_CH_2_OH, purity ≥99%, Sigma Aldrich) was added. The solution was emulsified using ultrasonication (Qsonica sonicators, Newtown borough, CT, USA) for 3 min with a 1 s on/off pulse at 30% amplitude. Subsequently, 10 mL of 3% (wt./vol.) poly(vinyl alcohol) (Mw = 89 000–98 000, Sigma Aldrich) in distilled water was added to the PCL-CPO solution and ultrasonicated for additional 5 min with a 1 s on/off pulse at 30% amplitude at room temperature. After ultrasonication, the solution was stirred to dry off the solvents at room temperature for 24 h and concentrated by centrifugation (Eppendorf 5702, Germany) at 10,000 rpm for 5 min. The microparticle pellet was washed three times with Dulbecco’s phosphate-buffered saline (DPBS, Gibco, Carlsbad, CA, USA) to remove any residual additives. The resultant pellet was lyophilized and stored in a dry and cold place until further use. As control, PCL particles were prepared without CPO employing the same protocol.

### Encapsulation Efficiency/Loading Capacity of CPO in HOGs:

Encapsulation efficiency and loading capacity of CPO in HOGs was determined as a percentage of the amount of CPO lost after every washing step to the initial CPO concentration used. Every wash performed was kept at 60 °C for 24 h to remove water, ethanol, and additives. Any residual PCL was removed by adding 3 mL DCM to the dried residue and stirred on a magnetic stirrer at 500 rpm for 30 min and filtered through a poly(vinylidene fluoride) membrane filter (durapore membrane, 0.22 μm pore size, USA). The mass of remained non-encapsulated CPO was measured. Percent encapsulation efficiency and percent loading capacity for CPO in HOGs were calculated as follows:

(3) $$\text{ Encapsulation Efficiency }(\text{\%})=\;\frac{\text{ Total CPO added - CPO lost in washing }}{\text{ Total CPO added }}\times 100$$

(4) $$\text{CPO loading capacity }=\frac{\text{ Encapsulated CPO }}{\text{ Final mass of HOCs }}\times 100$$

### Wettability of the Surface of CPO Containing PCL Microparticles:

5 mg of pristine PCL or CPO containing PCL microparticles were used to cover a stainless-steel surface. A 20 μL water droplet was placed on the specimen surface, which was microscopically imaged after 30 s and analyzed using ImageJ software (NIH, Bethesda, MD, USA) to quantify the contact angle between the surface and the water droplet.

### Particle Size Distribution of PCL, CPO, and HOGs:

For size distribution, 1 mg of CPO powder, PCL microparticles, or HOGs were dispersed separately in 10 mL of ethanol and vortexed for 5 min to obtain a homogenous solution. 2 mL of each solution was analyzed on particle size distribution using dynamic lighting scattering (DLS, Malvern Nano ZS ZEN3600).

### Microscopic and Elemental Analyses of Microparticles and Hydrogels:

The microstructure of the lyophilized samples of PCL, HOGs, and GelMA scaffolds with and without oxygen-generating agents (e.g., CPO or HOGs) was evaluated using high-resolution SEM (Zeiss MERLIN HR-SEM) and analyzed on microstructure and porosity using the image-based analysis software ImageJ. To enhance contrast and reduce charging effects, the samples were coated with a 5 nm layer of Pt/Pd alloy SEM analysis. Ultra-high resolution field emission SEM elemental mapping in the form of EDS analysis was performed on intact or FIB (Nova 600 Nanolab DualBeam) edged samples to evaluate the elemental distribution of carbon, calcium, and oxygen.

### Synthesis of GelMA:

Gelatin methacryloyl (GelMA) was synthesized as previously reported.^[42]^ Briefly, 10% (wt./vol.) of powdered gelatin (Sigma Aldrich, Type A, 300 bloom from porcine skin) was dissolved in DPBS (Gibco, USA) and stirred at 60 °C until its complete dissolution. To the mixture, 8% (vol./vol.) of methacrylic anhydride (Sigma-Aldrich) was gradually added while constantly stirring the mixture, representing a high methacryloyl substitution.^[43]^ The reaction was carried at constant stirring for 1 h at 60 °C and 500 rpm. Two volumes of pre-heated (60 °C) DPBS were added to the solution. This was followed by dialysis of the solution mixture using 12–14 kDa cutoff dialysis membranes (Thermo Fisher Scientific) against deionized water. Dialysis was carried out for 1 week to remove any salts and unreacted methacrylic anhydride, after which the solution was filtered, and placed at −80 °C to freeze before lyophilizing. At the end of lyophilization, freeze-dried GelMA was obtained in the form of white porous foam.

To determine the degree of methacrylation in the synthesized GelMA, fluoraldehyde o-phthaldialdehyde assay (OPA) was performed.^[44]^ Briefly, GelMA at 0.5 mg mL^−1^ was dissolved in DPBS. To this fluoraldehyde OPA reagent was added at a 1/1 (vol./vol.) ratio and allowed to react for 1 min to finish the reaction. Using a microplate reader, fluorescence intensity of the resulting mixture for conversion of amine groups in gelatin to methacrylated gelatin was read at 340 nm/455 nm. Gelatin as standard (*I*_standard_) and DPBS as blank (*I*_blank_) were used to calculate the degree of methyacrylation (DoM) by following the equation:

(5) $$\text{DoM}\;=1-\left(I_{\text{sample}\;}-I_{\text{blank}\;}\right)/\left(I_{\text{standard}\;}-I_{\text{blank}\;}\right)$$

### Mechanical Properties OMP Encapsulated GelMA Hydrogels:

The mechanical properties of particle-encapsulated GelMA hydrogels were conducted by a rotational parallel-plate rheometer (AR-G2 rheometer, TA instruments, USA) at room temperature. Disk shaped samples (n = 3 per group) with a thickness of 1.5 mm were placed between two 20 mm diameter plates and subjected to a frequency sweep at 0.1–10 Hz. The experiments were performed in a displacement-control mode for 1% shear strain. Storage (G′) and loss (G″) moduli were calculated against the applied frequency (Figure S6, Supporting Information).

### Expansion of hMSCs:

Bone marrow-derived Human Mesenchymal Stem Cells (hMSCs) that were purchased from Lonza (Allendale, NJ), were cultured in low glucose-DMEM media containing 10% fetal bovine serum, 100 IU mL^−1^ penicillin-streptomycin, and 1 ng mL^−1^ basic fibroblast growth factor (bFGF) in a humidified incubator at 37 °C with 5% CO_2_. Cell culture media was refreshed every third day. hMSCs were used for experiments between their 3rd and 5th passage.

### Fabrication of Self-Oxygenating Tissues:

10% (wt./vol.) GelMA was dissolved in DPBS solution at room temperature. Pristine CPO and HOGs were added to the GelMA solution at different concentrations (1%, 2.5%, 5%, 10%, and 20% (wt./vol.)). Additionally, 0.25% (wt./vol.) photoinitiator (Irgacure 2959, Ciba Specialty Chemicals) in DPBS was added to all polymer solutions to allow for UV-initiated photocrosslinking. The resulting solutions were poured into 8 mm wide and 1 mm thick cylindrical molds of polydimethoxysilane (Sigma Aldrich). The solutions were then exposed to UV light (60 s at 2.5 mW cm^−2^) to form disk-shaped self-oxygenating hydrogels. GelMA hydrogels without CPO or HOGs were used as controls. To create self-oxygenating tissues, GelMA hydrogels containing various concentrations of CPO and HOGs were prepared using low glucose-DMEM cell culture medium supplemented with 0.25% (w/v) photoinitiator. Trypsinized hMSCs were suspended in CPO-GelMA and HOG-GelMA prepolymer solutions at a cell density of 3 × 10^6^ cells mL^−1^. Cell-laden hydrogel precursor solutions (50 μL) were pipetted onto 3-(trimethoxysilyl) propyl methacrylate coated glass and crosslinked via UV light exposure (850 mW power with 8.5 cm working distance) for 40 s. The hydrogels were cultured in a bi-weekly refreshed DMEM-low glucose medium under normoxic (21% O_2_, 5% CO_2_, and 74% N_2_) and hypoxic (1% O_2_, 5% CO_2_, and 94% N_2_) culture conditions.

### Water Uptake of Hydrogel Constructs:

The weight of GelMA, CPO-GelMA, and HOG-GelMA was determined and represented as W_0_. Each sample was immersed in DPBS at 37 °C for 24 h. The hydrogel was gently removed from the DPBS solution and after blotting extra DPBS with paper tissue, it was weighed (W_t_). The swelling ratio (S.R.) of each sample was calculated according to the following equation:

(6) $$\text{S.R.}\;=\left({\text{W}}_{\text{t}}-{\text{W}}_{0}\right)/{\text{W}}_{0}\times 100$$

*W*_0_ is the initial weight of the hydrogel and *W*_t_ the weight of the hydrogel after 24 h of incubation.

### Quantification of Hydrogen Peroxide and Oxygen Release:

All experiments in hypoxia condition were performed by a hypoxic chamber (Stem Cell Technology, USA), with the O_2_ concentration controlled by an electrode-based ISO-OXY 2 oxygen sensor (World Precision Instruments) and maintained at 2–3%. The closed-system of oxygen chamber could not exchange any material, oxygen, or matter with the surroundings (Figure S1, Supporting Information).

GelMA hydrogel (1 mL) constructs with different amounts of CPO and HOGs (0%, 1%, 2.5%, 5%, 10%, and 20% (wt./vol.) were individually placed into the wells of a 24-well plate containing 3 mL of deoxygenated DPBS for up to 12 days. 50 μL of media was collected daily and quantitated on its hydrogen peroxide concentration using an Amplex red hydrogen peroxide assay kit (Thermo Fisher Scientific, USA) in the presence of peroxidase from Horseradish type VI (HRP, Sigma Aldrich, 250 U mg^−1^, Sigma-Aldrich) according to the manufacturer’s protocol. To this, the mixture of 100 μm Amplex red and 0.25 U mL^−1^ HRP in distilled water was prepared. Since Amplex red reagent is not able to detect exact concentration of hydrogen peroxide below 10 μm, plasma-treated CPO- and HOGs-laden hydrogels were diluted 200 times before adding to reagent. 50 μL of the Amplex Red/HRP reagent was mixed with 200 μL of the 200×-diluted plasma-treated samples in a 96-well plate and incubated for 1 h. The resulting fluorescence signal was determined out using a Tecan well-plate reader (BioTek instruments, Ink, USA) using *λ*ex = 560/20 nm for excitation wavelengths and *λ*em = 590/20 nm for emission wavelengths. To determine the oxygen generation profiles, 24 well plate containing GelMA hydrogel constructs with different amounts of CPO and HOGs (0%, 1%, 2.5%, 5%, 10%, and 20% (wt./vol.) in the GelMA solution) were placed in a near-airtight glovebox (Stem Cell Technology, USA) that was continuously purged with nitrogen gas (Figure S1, Supporting Information). The O_2_ concentration of the media within the 24 well plates was measured using an electrode-based ISO-OXY 2 oxygen sensor (World Precision Instruments) for up to twelve days.

### Cell Survival in CPO-GelMA and HOGs-GelMA Hydrogels:

To evaluate cell survival, 1 × 10^6^ hMSCs were encapsulated in 1 mL of 10% (w/v) GelMA hydrogels. Cell viability was evaluated by staining hMSCs encapsulated in GelMA hydrogel using Live/Dead assay (Invitrogen, USA) according to the manufacturer’s instructions after 1, 3, 6, and 12 days of culture. Stained cells were visualized and microphotographed using fluorescence microscopy (Zeiss, Axio Observer A.1). The metabolic activity of encapsulated cells was assessed using PrestoBlue assay (Invitrogen, USA) according to the manufacturer’s protocol and measured using a BioTek Synergy 2 plate reader.

### Simulations for Oxygen Release:

Numerical simulations were performed based on a previously validated multiscale in silico model of bone fracture healing.^[45–46]^ The hybrid framework combines partial differential equations at the tissue level to model the key processes of tissue formation and implant integration. The hybrid framework is based on an agent-based description at the cellular level that simulates developing vasculature with discrete endothelial cells, including the intracellular Dll4-Notch signaling in every endothelial cell.^[46]^ The development of discrete vascular trees, which serve as a nutrient source, is determined by sprouting, vascular growth followed by anastomosis at the cellular level. The sprouting of host vasculature is modeled by capturing the intracellular levels of VEGFR-2, active VEGFR-2, effective active VEGFR-2, Notch1, active Notch1, effective active Notch1, Dll4, and actin. The rules that capture the lateral inhibition mechanism during tip cell selection were adapted from a previously developed agent-based model.^[47]^ At the tissue level, the tissue formation and implant integration is described as a spatiotemporal variation of 10 continuous variables, which was previously described for simulation of the bone fracture healing process.^[45]^ In short, the considered cell types (i.e., progenitor cells and fibroblasts) can migrate, proliferate, and secrete growth factors (i.e., VEGF). Tissues, nutrients, and blood vessels are modeled in separate spaces and can thus “co-exist” in the same location. Here, we adapted the previously developed model (36) by changing the geometry, the boundary conditions, and the VEGF-production rate of the fibroblasts (non-dimensional of 10 in comparison to 1 previously) to emulate the oxygen-beads set-up. CPO and HOGs were modeled as oxygen sources, adapting the generation rate to approximate the experimentally measured oxygen generation kinetics of the CPO or HOGs:

(7) $$Bead\;release\;=\frac{G_{n}\cdot H_{n}^{6}}{n^{6}+H_{n}^{6}}\cdot e^{-\tau .t}$$

with non-dimensional values *G*_*n*_ = 2.2, *H*_*n*_ = 0.11, and *τ* = 0.5 for CPO and *τ* = 0.05 for HOGs. The beads were randomly positioned in the simulation domain at 1%, 2%, or 4% of the simulation area and the angiogenic sprouts could grow into the simulated tissue implant from all sides (Figure S7, Supporting Information). Progenitor cells and fibroblasts could also migrate into the tissue implant from the surrounding tissues (non-dimensional Dirichlet boundary condition of 0.01 during the first 3 days). The implant was initialized at 3.7% oxygen tension, fibrous matrix (non-dimensional IC_mf_ = 0.1), and progenitor cells at 10% of 10% of the carrying capacity (non-dimensional ICmsc = 0.1). All simulations were run multiple times to account for the stochastic bead placement and were checked for consistency. The simulation code is available upon request.

### VEGF and DNA Quantifications for Angiogenesis:

To determine the amount of VEGF secreted by the engineered tissues, GelMA hydrogels containing 3·× 10^6^ cells/mL of hMSCs and 2.5% HOGs were cultured under anoxic conditions while GelMA hydrogels without HOGs were cultured under normoxic, hypoxic, and anoxic conditions. All hydrogels were cultured in DMEM low-glucose media containing 10% fetal bovine serum and 100 IU mL^−1^ penicillin-streptomycin for six days. After 1, 3, and 6 days of culture, the media’s VEGF levels were quantitated using a Human VEGF ELISA Kit (BioAim Scientific Inc., Cat. No: 1 010 015) according to manufacturer’s instructions. The amount of VEGF was normalized for the hydrogel’s DNA content, which was quantified using the CyQuant kit (Invitrogen, Cat. No: C7026) according to the manufacturer’s instructions.

### Subcutaneously Implantation of Self-Oxygenation Tissues:

To evaluate the in vivo behavior of self-oxygenating tissues, hydrogels composed of various concentrations (0%, 1%, or 2%) of HOGs, 2 × 10^6^ cells mL^−1^ of hMSCs, and 10% (w/v) GelMA were fabricated. Hemispherical hydrogels (1 mm thickness and 8 mm diameter) were subcutaneously implanted in the backs on 12-week old nude rats (Charles River) based on a previously established protocol (2017N000114) by the Institute’s Committee on Animal Care. Briefly, anesthesia was induced and maintained with isoflurane in spontaneously breathing rats. After seven days, the implants were surgically extracted and frozen in OCT compound. The frozen samples were sectioned in 5 μm using a cryomicrotome. Sections were stained with TUNEL assay, DAPI based cell quantification, CRP, and HNA to assess survival and hypoxic stress of hMSCs. Also, vascular network formation was analyzed by staining for CD31. Evaluation of sample integration and degradation was performed by H&E and Masson trichrome stain. All stained slides were examined using a Nikon inverted microscope (TS2-LS, Tokyo, Japan).

To evaluate the number and diameter of vessels in the explants, three power field images (40×) were taken alongside the borders and inside of the hydrogels. Representative images were then evaluated by two blinded independent analysts using the software Image J (National Institutes of Health, United States). The number of vessels was counted by using the following inclusion criteria: Only the presence of a visible lumen associated with positively labeled CD31 cells was counted as vessels, vessels presenting in the outer border of the high-power field were not counted, structures positively stained with CD31 but without visible lumen were discarded. Staining artifacts were identified by CD31 staining not associated with DAPI co-staining of endothelial cell nuclei; these artifacts were also discarded. The number of vessels per high power field was expressed by the mean number of vessels counted by the two analysts in each representative image. Additionally, to measure vessel diameter, the transversal diameter of counted vessels was measured by using the software Image J, and the diameter of vessels was expressed by the mean of vessel diameter measured by the two analysts in each of the representative images.

### Statistical Analysis:

For statistical relevance, all experiments were performed using, at least, triplicates. All data has been presented by mean ± standard deviation (SD) or standard error mean in each experiment. ANOVA, *t*-test, and Duncan’s new multiple-range test was used for statistical analysis where appropriate.

## Supplementary Material

supinfo
