# Supplementary material for: Self-Oxygenation of Tissues Orchestrates Full-Thickness Vascularization of Living Implants
Source: Adv Funct Mater. Author manuscript; Available in PMC 2022 Oct 14. (PMC8680410; doi:10.1002/adfm.202100850)
Supplement: supinfo [file NIHMS1722155-supplement-supinfo.pdf]

© 2021 Wiley-VCH GmbH

ADVANCED FUNCTIONAL MATERIALS

Supporting Information

for *Adv. Funct. Mater.,* DOI: 10.1002/adfm.202100850

Self-Oxygenation of Tissues Orchestrates Full-Thickness Vascularization of Living Implants

*Ali Farzin, Shabir Hassan, Liliana S. Moreira Teixeira, Melvin Gurian, João F. Crispim, Varun Manhas, Aurélie Carlier, Hojae Bae, Liesbet Geris, Iman Noshadi, Su Ryon Shin, and Jeroen Leijten**

Supporting Information

Self-oxygenation of Tissues Orchestrates Survival and Full-thickness Vascularization of Living Implants

Ali Farzin[1,2,‡], Shabir Hassan[1,‡], Liliana S. Moreira Teixeira[3], Melvin Gurian[3], João F. Crispim[3], Varun Manhas[4], Aurélie Carlier[5], Hojae Bae[6], Liesbet Geris[4], Iman Noshadi[7], Su Ryon Shin[1], and Jeroen Leijten[1,3,]*

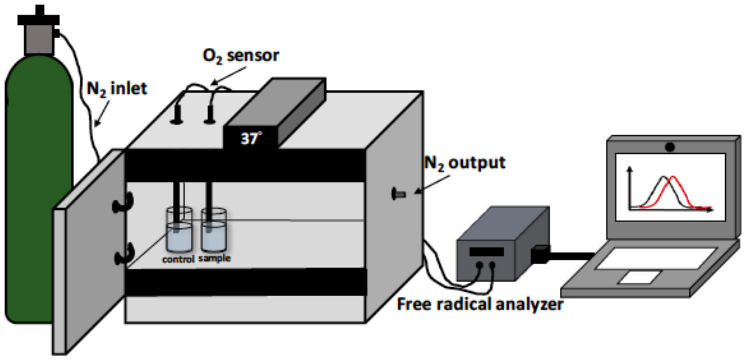

Figure S1: Schematic illustration of hypoxia chamber for the evaluation of oxygen and hydrogen peroxide release and cell behavior of developed HOGs.

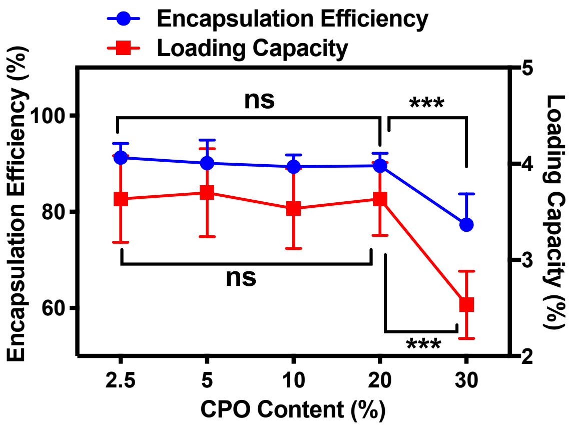

Figure S2. Encapsulation efficiency and CPO loading capacity in HOGs at different CPO (wt./wt.) concentrations. Significant differences are shown as; ns, non-significant, p*** < 0.001.

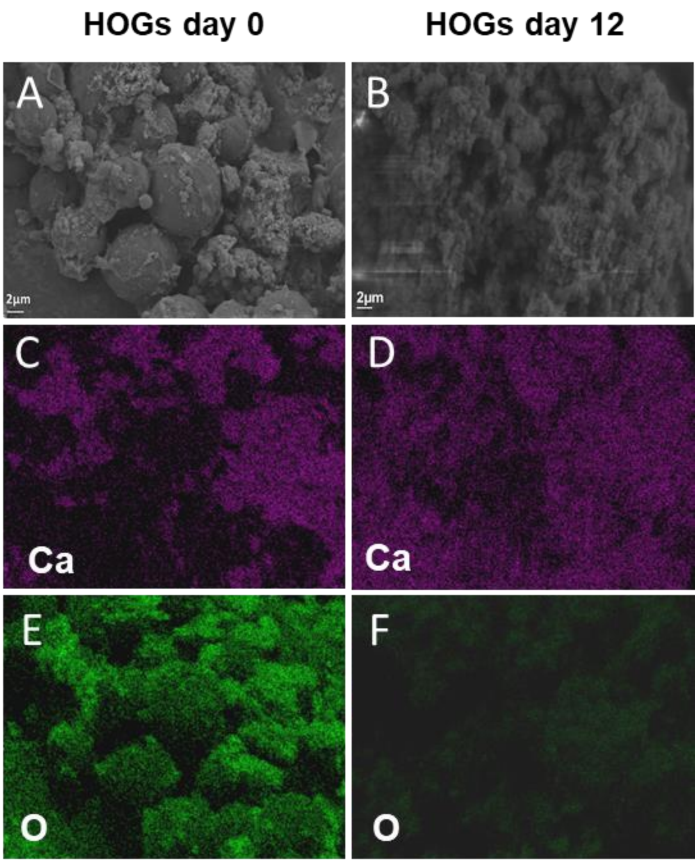

Figure S3. (A, B) SEM images and elemental mapping of (C, D) calcium and (E, F) oxygen by EDS of HOGs before and after 12 days of immersion in DPBS solution.

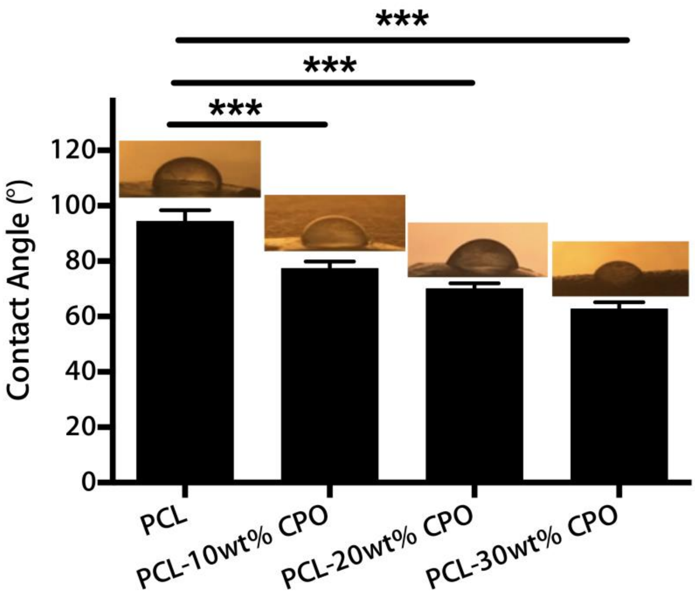

Figure S4. Water contact angle analysis of PCL and three formulation of HOGs with distinct amounts of CPO content. Significant differences are shown as p*** < 0.001.

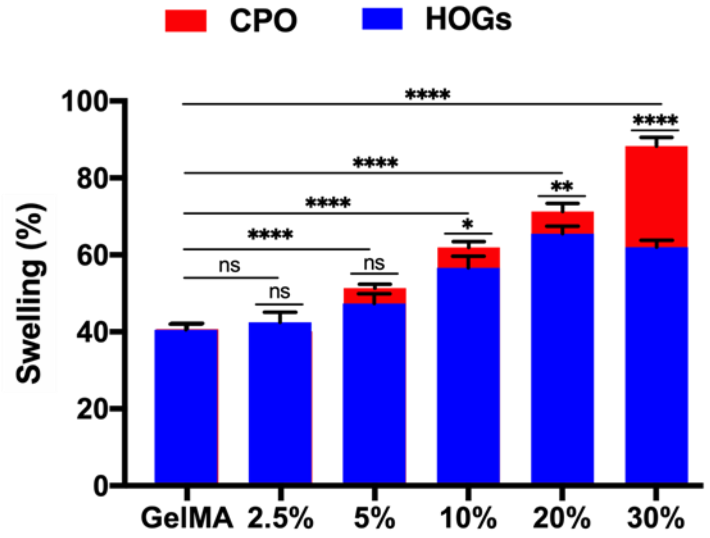

Figure S5. Swelling ratio of GelMA hydrogel containing distinct concentrations of CPO and HOGs. Two-way Anova, multiple comparisons. Significant differences are shown as; ns, non-significant, p* < 0.05, p** < 0.01, p**** < 0.0001.

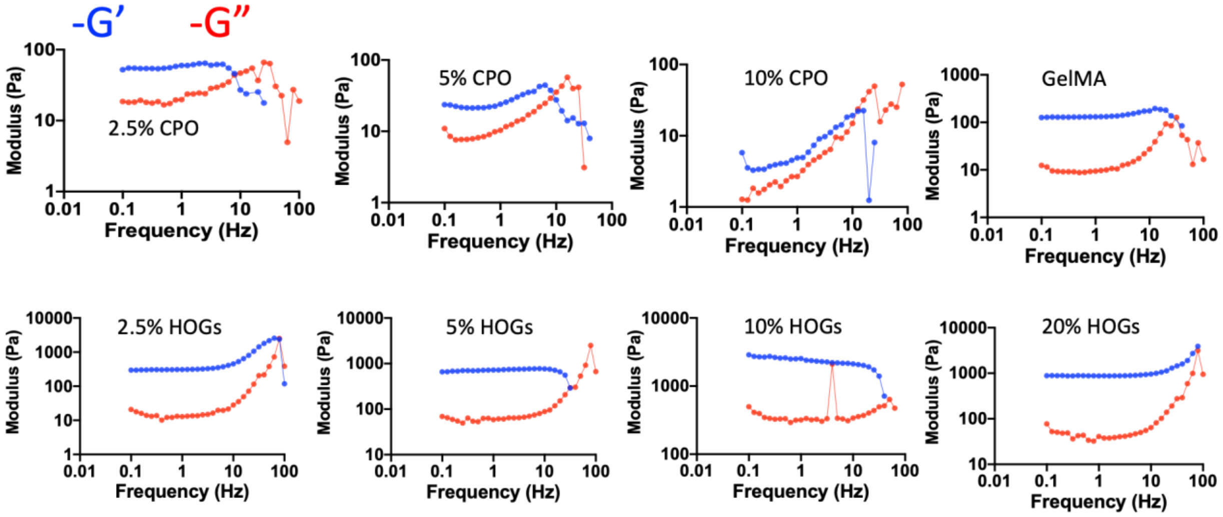

Figure S6. Storage (G') and loss (G'') moduli for GelMA hydrogels with different concentrations of CPO and OMPs.

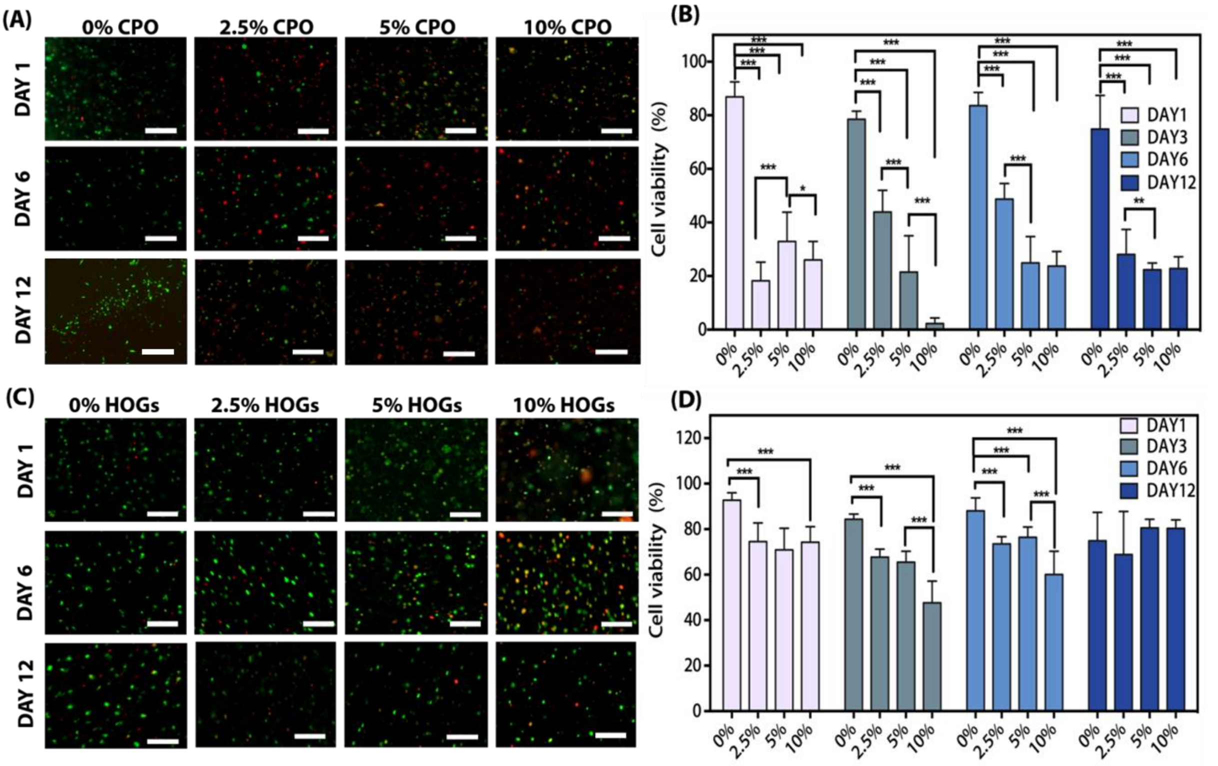

Figure S7. Cell viability with live (green) and dead (red) hMSCs in (A, B) CPO-based and (C, D) HOGs-based oxygen generating GelMA hydrogels under normoxic culture conditions as determined with (A, C) confocal fluorescent microscopy and (B, D) associated semi-quantitative image analysis (n =7). Scale bars equal 100 µm. Significant differences are shown as *p < 0.05, **p < 0.01, and ***p < 0.001.

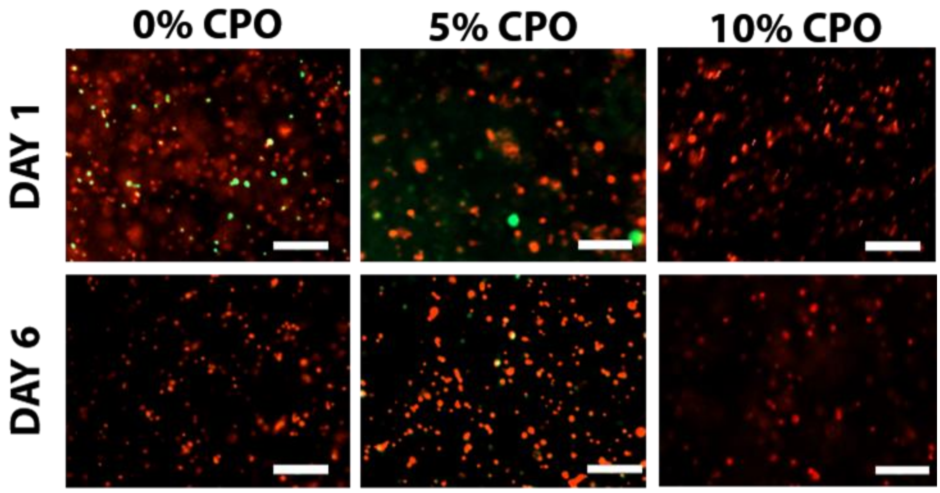

Figure S8. Microphotographs of live (green) and dead (red) stained MSCs in GelMA hydrogel containing different concentrations of CPO under anoxic culture conditions. Scale bars equal 100 µm.

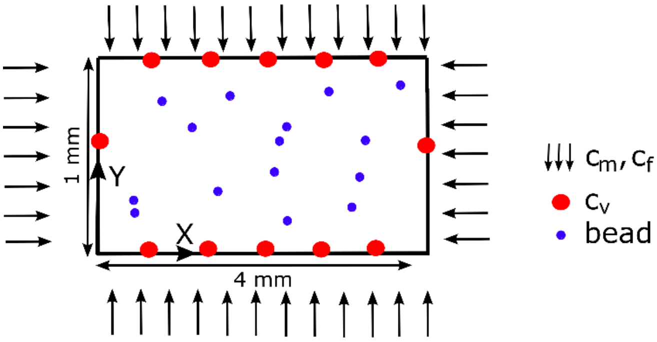

Figure S9. Schematic overview of the modelled implant. At the start of the simulation the modelled implant was filled with loose fibrous tissue matrix and randomly placed oxygen-generating beads. The Dirichlet boundary conditions show the starting points of the blood vessels (cv) and the sites of release of progenitor cells (cm) and fibroblasts (cf) after implantation.

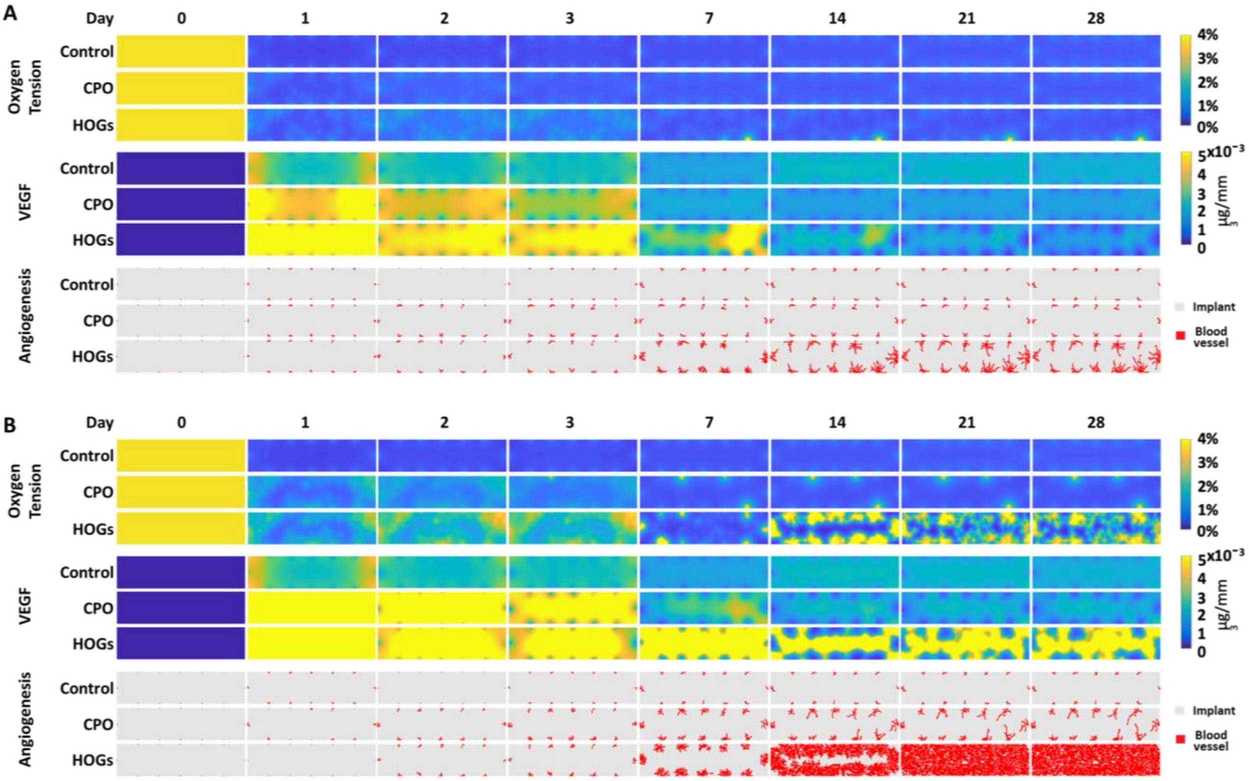

Figure S10. Simulated numerical model of oxygen tension, VEGF release, and angiogenesis (implant is indicated in grey and ingrowing blood vessels are indicated in red) in non-oxygenating GelMA hydrogel and GelMA hydrogel containing (A) 1 or (B) 4 % (wt./vol.) % of either CPO or HOGS following virtual implantation.
